# Annotations

**Published:** 1889-08-10

**Authors:** 


### Annotations.

**Elixirs of Life.**

"Dr. Brown-Sequard's elixir of life is beaten by a spring in Nevada which is said to have turned a septuagenarian negro into a brisk and frolicsome youth." Thus does the common-sense of mankind laugh at the extravagances of specialists when they forget the limitations which reason and experience impose upon them. Dr. Brown-Sequard is now an old man. He has in his day done much good physiological work, and, of course, some that has not been particularly good. He was at his best when experimental physiology was comparatively a new art; and no doubt, like many others, was often somewhat intoxicated with the wonderfulness of his discoveries and confirmations. It is not therefore surprising that in his old age, when wonders are no longer either so numerous or so easy of discovery in the physiological field, he should try to resuscitate both his fame and his failing energies by methods new and strange. All the world knows of the decoction which he made and injected into his aged blood, and the surprising effects which are said to have been produced. And all the world, at least the world that has not yet divested itself of reason, has shaken its head and felt sorry for the doting old man, who hoped by such means to indefinitely postpone the inevitable. Elixirs of life innumerable have been discovered and believed in; but seldom before has any member of the medical profession exhibited himself on a prominent stage as the hero of a successful experiment in the doubtful art. Truth to say, there are no such things as "elixirs of life," nor are there any decoctions of any kind which will preserve health and secure a green old age. There are ways of regaining health when lost, of preserving it when regained, and of prolonging life to a longer than ordinary period. But these ways do not pass through the drug shop or the elixir maker's arcana. They follow the beaten tracks of moderate eating, drinking, and working, of sufficient physical exercise, of open-air work and pleasures, of cheerfulness, honesty, and content. If Dr. Brown-Sequard represents the "drug and decoction" method of healthy age and long life, Mr. Gladstone may very well stand for the type of the healthy age and long life which physical exercise, moderation in eating and drinking, and fresh air can produce. If it be asked whether of the two is the more vigorous specimen, everybody will know how to answer. In physical and mental energy Mr. Gladstone at eighty has seldom been equalled, and, perhaps, hardly ever surpassed. It is fortunate for the world at large that a healthy and happy old age does not depend upon "drugs and decoctions," but upon "rational methods of living." For thus a healthy old age is possible for most people who have the good sense and the strength of mind to learn and to practice the simple arts of healthy life. All ordinary persons may secure that comfortable longevity which is so eminently desirable if they will practice what nature and experience preach. But if they will not do this, all the decoctions of all the physiologists in the world will not avail them.

**A "Salvation Army" Hospital.**

It is stated by a morning contemporary that the Salvation Army contemplates the establishment of a new hospital in the metropolis. We are not aware how far this statement may be founded in fact, but there is no doubt that if a hospital were erected in a suitable district and financed and managed by General Booth and his followers a most interesting series of experiments would be made. Probably no public work has ever been carried on, at any rate in recent times, which has been at once so successful and economical as the work of the Salvation Army. Whatever individual opinion may be of the value of the work and of the nature of the methods employed, there cannot be any diversity of judgment as to the results achieved and the smallness of the cost. General Booth, a mere private individual, and we believe an obscure Methodist preacher twenty-five years ago, has not only created a veritable army of striking magnitude, but has sent portions of it, conquering and to conquer, into every part of the world. And what were the elements out of which this extraordinary army has sprung? "The rabble," many people would say. But if not the rabble, at least to a large extent the poor, the uneducated, and the unknown have awakened as from a sleep of death at the trumpet-call of General Booth, and have done, and are doing, the work of a disciplined army. Few will deny that there is now as great an opportunity for a striking display of efficiency and economy in the hospital world as there was in the greater world twenty-five years ago. Most people will agree that it would be difficult to find a more suitable man for the experiment than General Booth. For our part, much as we deprecate the increase of hospitals at the present time, we should waive all objections in favour of an attempt by so unique a personality as the leader of the Salvation Army. We hope the report is true, and that the experiment will be made with as little delay as possible. Nothing is so effective a teacher as an object lesson; and an object lesson of a very striking character may undoubtedly be looked for when the first annual report of the Salvation Army's hospital makes its appearance.

**Gipsy and Van Children.**

It will probably come as a surprise to many to be informed that in England and Scotland there are no fewer than fifty thousand gipsy and van children. That those children are for the most part brought up in a wild and semi-savage way, and that they are almost entirely destitute of education, will, however, surprise nobody who has ever lived long enough in the country to understand their ways of life. In a populous country like ours there is, and always must be, very much public work to be done of a thankless and discouraging nature. But, happily for us, our intentions are at least good, and our resources are by no means limited. Where public energy and zeal fail private philanthropy steps in, and very often does more and better work than any public functionaries could be made to accomplish. When this is the case, as it frequently is, the least the Legislature can do is to give all possible furtherance and help to the private individuals who thus take upon themselves the labour, worry, and cost which the public should undertake. But, thanks to the stupidity and inconsequence of many members of Parliament, whose tempers are as wilful as their brains are apparently feeble, it not seldom happens that such philanthropists are thwarted, hindered, worried, and rendered powerless in a thousand different ways. Any cackling goose, for example, can block a Bill in Parliament; and thus, session after session, the most valuable and necessary measures may be indefinitely deferred. Mr. George Smith, of Coalville, whose name is a household word wherever canal boats and canal children are known and spoken of, is endeavouring to do for the 50,000 van and gipsy children of England and Scotland what has already been done, chiefly through his persistent efforts, for the 30,000 canal children. These latter are now brought under the influence of the Elementary Education Act, and will thus have at least a chance of becoming instructed and responsible men and women. But Mr. Smith's efforts are persistently frustrated by the action of three or four "blockers" in the House of Commons, and thus, in spite of all his efforts, session after session passes by and nothing is done. Mr. Smith publishes a "solemn protest" against what he calls the "trifling action of the blockers," and affirms that a "tremendous responsibility rests upon them for keeping the poor children in ignorance." Undoubtedly Mr. Smith is justified in the energy of his language. There can be no sufficient reason for delay or for withholding from 50,000 van and gipsy children what has already been done with so much advantage on behalf of 30,000 canal children. It is exceedingly to be desired that all who can bring influence to bear upon the House of Commons as a whole, or upon individual members thereof, will come to Mr. Smith's help in his noble, philanthropic, and urgently needed work.

